# First Complete Genome Sequences of *Xanthomonas citri* pv. vignicola Strains CFBP7111, CFBP7112, and CFBP7113 Obtained Using Long-Read Technology

**DOI:** 10.1128/genomeA.00813-17

**Published:** 2017-09-07

**Authors:** Mylène Ruh, Martial Briand, Sophie Bonneau, Marie-Agnès Jacques, Nicolas W. G. Chen

**Affiliations:** IRHS, INRA, Agrocampus Ouest, Université d’Angers, SFR4207 QUASAV, Beaucouzé, France

## Abstract

*Xanthomonas citri* pv. vignicola strains cause bacterial blight of the legume crop cowpea. We report whole-genome sequences of three *X. citri* pv. vignicola strains obtained using PacBio single-molecule real-time sequencing. Such genomic data provide new information on pathogenicity factors, such as transcription activator-like effectors.

## GENOME ANNOUNCEMENT

Cowpea (*Vigna unguiculata*) is a legume crop grown in semiarid tropical and subtropical regions of Africa, America, and Asia (1). It is used as staple food in different forms (grain, green pods, roots, or leaves) and as animal feed (2). Cowpea bacterial blight (CoBB) is a major disease of cow pea that severely reduces crop yields (3) and is present wherever cowpea is grown (4). The causal agent of CoBB is *Xanthomonas citri* pv. vignicola (formely *X*. *Axonopodis* pv. vignicola) (5). *X. Citri* pv. vignicola mainly produces symptoms on leaves, starting as tiny water-soaked spots that evolve into large necrotic areas surrounded by a yellow halo. This pathogen can also cause stem canker and water-soaking of pods, providing the opportunity for the bacteria to gain access to seeds (6). CoBB is seed-borne and can also be transmitted by aerosols and insects (7). Moreover, *X. citri* pv. vignicola is able to survive in infected plant debris, seeds, and soil, which constitute different inoculum sources (8, 9). Some genotypes of cowpea have been selected for resistance to CoBB (10); however, no information on the molecular basis for this resistance is available to date.

We sequenced three strains of *X. citri* pv. vignicola using PacBio single-molecule real-time (SMRT) sequencing (11). Genomic DNA was extracted using the Wizard genomic DNA purification kit (Promega) according to the manufacturer’s recommendations. Whole genomes were sequenced using the PacBio RSII SMRT technology at the Icahn School of Medicine at Mount Sinai (New York, NY). One SMRT cell was used per strain to achieve ~100× coverage. *De novo* assembly was performed using Canu version 1.5 (12), circularization of the contigs was done using Circlator version 1.5.1 (13), and polishing was done using the Quiver algorithm (14). Annotation was performed with the NCBI Prokaryotic Genome Annotation Pipeline (15).

Each *X. citri* pv. vignicola genome comprised one chromosome plus zero to three circularized plasmids per strain. The genome of strain CFBP7111 comprised three plasmids, for a total assembly size of 5,710,499 bp, with a G+C content of 64.5% and a total of 5,095 predicted coding sequences (CDS). The genome of strain CFBP7112 comprised two plasmids, for a total assembly size of 5,155,996 bp, with a G+C content of 64.7% and a total of 4,460 predicted CDS. The genome of strain CFBP7113 comprised no plasmids, for a total assembly size of 5,105,205 bp, with a G+C content of 64.7% and a total of 4,446 predicted CDS. Interestingly, the content and localization of genes encoding transcription activator-like type III effectors (*tal*) differed among the three genomes. Strain CFBP7111 had no *tal* gene, while strain CFBP7112 contained seven *tal* genes located on plasmids, and strain CFBP7113 bore one *tal* gene located on the chromosome. This diversity may reflect different modes of adaptation of these strains to cowpea.

### Accession number(s).

The three whole-genome sequences have been deposited at GenBank under accession numbers CP022263 to CP022266 for strain CFBP7111, CP022267 to CP022269 for strain CFBP7112, and CP022270 for strain CFBP7113.
